# Effect of the Biofilm Age and Starvation on Acid Tolerance of Biofilm Formed by *Streptococcus mutans* Isolated from Caries-Active and Caries-Free Adults

**DOI:** 10.3390/ijms18040713

**Published:** 2017-03-30

**Authors:** Shan Jiang, Shuai Chen, Chengfei Zhang, Xingfu Zhao, Xiaojing Huang, Zhiyu Cai

**Affiliations:** 1 Department of Endodontics and Operative Dentistry, School and Hospital of Stomatology, Fujian Medical University, 246 Yangqiao Zhong Road, Fuzhou 350002, China; kqshanj@163.com (S.J.); drchenshuai@163.com (S.C.); 2 Department of Endodontics, Comprehensive Dental Care, Faculty of Dentistry, The University of Hong Kong, Hong Kong, China; zhangcf@hku.hk; 3 Department of Endodontology, Tianjin Stomatological Hospital of Nankai University, 75 North Dagu Road, Heping District, Tianjin 300000, China; xingfu_108@126.com; 4 Department of Oral and Maxillofacial Surgery, Fujian Medical University Union Hospital, Fuzhou 350001, China; caizhiyu2007@126.com

**Keywords:** *Streptococcus mutans*, biofilm formation, aciduricity, Starvation, confocal laser scanning microscopy

## Abstract

*Streptococcus mutans* (*S. mutans*) is considered a leading cause of dental caries. The capability of *S. mutans* to tolerate low pH is essential for its cariogenicity. Aciduricity of *S. mutans* is linked to its adaptation to environmental stress in oral cavity. This study aimed to investigate the effect of biofilm age and starvation condition on acid tolerance of biofilm formed by *S. mutans* clinical isolates. *S. mutans* clinical strains isolated from caries-active (SM593) and caries-free (SM18) adults and a reference strain (ATCC25175) were used for biofilm formation. (1) Both young and mature biofilms were formed and then exposed to pH 3.0 for 30 min with (acid-adapted group) or without (non-adapted group) pre-exposure to pH 5.5 for three hours. (2) The mature biofilms were cultured with phosphate-buffered saline (PBS) (starved group) or TPY (polypeptone-yeast extract) medium (non-starved group) at pH 7.0 for 24 h and then immersed in medium of pH 3.0 for 30 min. Biofilms were analyzed through viability staining and confocal laser scanning microscopy. In all three strains, mature, acid-adapted and starved biofilms showed significantly less destructive structure and more viable bacteria after acid shock than young, non-adapted and non-starved biofilms, respectively (all *p* < 0.05). Furthermore, in each condition, SM593 biofilm was denser, with a significantly larger number of viable bacteria than that of SM18 and ATCC25175 (all *p* < 0.05). Findings demonstrated that mature, acid-adapted and starvation might protect biofilms of all three *S. mutans* strains against acid shock. Additionally, SM593 exhibited greater aciduricity compared to SM18 and ATCC25175, which indicated that the colonization of high cariogenicity of clinical strains may lead to high caries risk in individuals.

## 1. Introduction

Dental caries is an infectious and transmissible bacterial disease [1,2]. *Streptococcus mutans* (*S. mutans*) has been described as the principal etiologic agent of human dental caries and an important constituent of dental plaque [3]. *S. mutans* varies in cariogenicity as a result of different growing conditions such as carbohydrate source, pH, biofilm presence, starvation, and slight variations in different species [4]. It is widely accepted that strong capability of *S. mutans* to tolerate low pH, namely aciduricity, is essential for its cariogenicity [5]. The constitutive acid tolerance properties of *S. mutans*, as well as its adaptive acid tolerance response (ATR) provide a holistic benefit to withstand continual changes in acid shock [6]. It has been reported that ATR plays a crucial role in protecting *S. mutans* from more severe acid stress [7]. In other words, acid adaption triggered by prior exposure to low but nonlethal pH enables *S. mutans* to survive in severe acidification.

Moreover, biofilm has significant effects on the aciduricity of the bacteria because of the complexity of the oral environment [8]. Several studies have shown that *S. mutans* in biofilms have increased adaptive capabilities to adverse conditions and display increased tolerance to antimicrobial treatments [9,10]. For example, biofilm cells were tremendously more tolerant to acid stress, with a 41.5% survival rate, while such rate was only 0.00004% for planktonic cells [11]. Interestingly, starvation also has been suggested to induce the tolerance of planktonic bacteria to acid shock [12]. Thus, bacteria in oral biofilm have been speculated to be more tolerant to acid during the starvation between meals [13].

On the other hand, it is worth mentioning that *mutans streptococcus* (MS) are found in all peoples regardless of race, ethnic background or geographic origin, with *S. mutans* (serotype c) as majority. Normally, *S. mutans* is present in the mouth as an insignificantly small component of oral flora. When people have bad oral hygiene and unhealthy diet for a long time, *S. mutans* may become a dominant member in plaque flora and cause caries [14]. However, the amount of *S. mutans* is not always parallel with caries level [15]. A growing body of evidences supports that even the same serotype c *S. mutans* clinical isolates could be genetically different [16,17]. Additionally, how these differences affect virulence properties of cariogenic *S. mutans* clinical isolates is still poorly understood.

To answer these questions, we previously examined one patient with multiple active carious lesions, and the level of *S. mutans* in his saliva was not high enough to explain his situation. We then acquired a plaque sample from his healthy smooth enamel surface, not from carious lesions, considering that *S. mutans* are most strongly associated with the onset of caries [18,19,20]. Using polymerase chain reaction (PCR), we identified five *S. mutans* (serotype c) clinical isolates from this sample. We found that both their genotypes and virulence properties were different. One of the clinical isolates, named SM593, has already been isolated previously from the caries-active adult. Compared with SM18 from the caries-free adult, SM593 grew faster and was more competent in adhesion and biofilm formation [21,22,23,24]. However, limited information is now available on the differences in the aciduricity of their biofilms under different conditions.

In the present study, we examined the biofilms of *S. mutans* clinical strains SM593 and SM18 and the standard strain ATCC 25175 in terms of aciduricity in different conditions including biofilm formation in different phases (young and mature biofilms), acid adaptation (acid-adapted and non-adapted biofilm) and starvation (starved and non-starved biofilms) using confocal laser scanning microscopy (CLSM) with molecular probes LIVE/DEAD BacLight viability stain.

## 2. Results

### 2.1. Effect of ATR on Acid Tolerance of S. mutans Biofilm

To determine the effect of ATR on acid survival of biofilm cells of *S. mutans*, acid adaptation experiment was conducted by exposing biofilms to pH 5.5 for 3 h and then exposing to pH 3.0 for 30 min (acid-adapted group), and exposing biofilms to pH 3.0 for 30 min without prior acid adaptation as comparative controls (non-adapted group). The differences in morphological feature and viability of biofilms were compared and summarized as following.

#### 2.1.1. Differences in Acid Tolerance between the Acid-Adapted and Non-Adapted Biofilms

**Figure 1:**
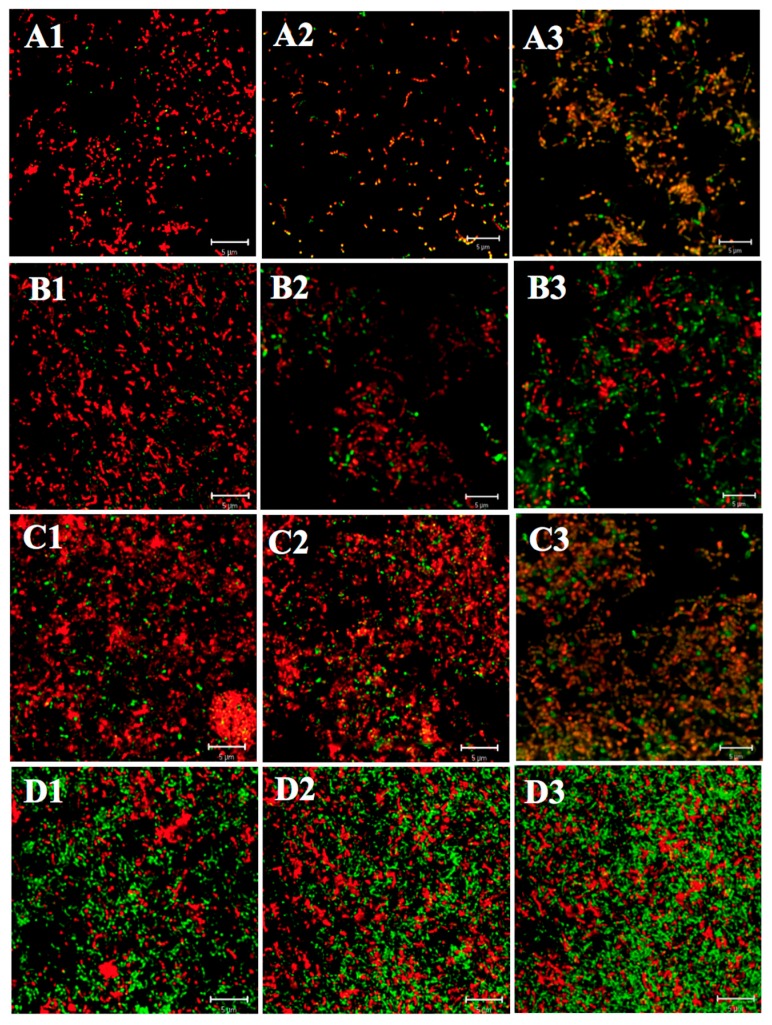
Confocal laser scanning microscopy (CLSM) images of Live/Dead (SYTO-9 and PI) stained young and mature biofilms treatment with acid-adapted or non-adapted. Young biofilm in non-adapted group (**A1**), ATCC25175; (**A2**), SM18; (**A3**), SM593; Young biofilm in acid-adapted group (**B1**), ATCC25175; (**B2**), SM18; (**B3**), SM593; Mature biofilm in non-adapted group (**C1**), ATCC25175; (**C2**), SM18; (**C3**), SM593; Mature biofilm in acid-adapted group (**D1**), ATCC25175; (**D2**), SM18; (**D3**), SM593 (scale bars, 5 mm; magnification 630×).

As shown in Figure 1, both young and mature biofilms of all three strains (ATCC 25175, SM18, and SM593) in the non-adapted group were dominantly composed of dead cells (red color), whereas young and mature biofilms of all three strains in the acid-adapted group were composed mainly of live cells (green), forming distinct network shapes. Additionally, as shown in Table 1 and Table 2, the viability in each layer (inner, middle and outer) of young and mature biofilms in the acid-adapted group was significantly higher than that of in the non-adapted group (all *p* < 0.05). Lastly, the differences between three different layers were compared. In general, the order of the viability among three layers was: middle layer > inner layer > outer layer. Specifically, within each group, the number of viable biofilms cells in the middle layer was significantly more than that of in the outer and inner layers (all *p* < 0.05), and no significant difference was found between inner and outer layers (all *p* > 0.05).

#### 2.1.2. Differences in Acid Tolerance between Young and Mature Biofilms

To assess whether the biofilm age influences the aciduricity of biofilm cells of *S. mutans*, the viability and morphology of young and mature biofilms (formed after 3 h and 24 h incubation, respectively) were compared. For all tested strains, in both the acid-adapted and non-adapted groups, structure disruption of young biofilms was greater than that of mature biofilms (Figure 1), and the viability rates for cells in each layer of mature biofilms were significantly higher than those of young biofilms (Table 1 and Table 2) (all *p* < 0.05).

#### 2.1.3. Differences in Acid Tolerance on ATR among Three Strains

As seen in Figure 1, in either the acid-adapted or non-adapted group, the architecture of both young and mature biofilms formed by SM593 appeared to have relatively less loss of integrity than that of SM18 and ATCC 25175. In addition, as shown in Table 1 and Table 2, the viable cells in each layer of young and mature biofilms formed by SM593 were significantly more than those by SM18 and ATCC 25175 (all *p* < 0.05), while there was no statistical difference between SM18 and ATCC 25175 biofilms (all *p* > 0.05).

### 2.2. Effect of Starvation on Acid Tolerance of S. mutans Biofilm

To examine the effect of starvation on aciduricity of biofilm cells of *S. mutans*, starvation experiment was performed as described in the Materials and Methods section. Comparison of architecture and viability of starved biofilm (formed by incubating with PBS for 24 h) and non-starved biofilm (formed by incubating as normal) exposed to pH 3.0 for 30 min were made.

#### 2.2.1. Differences in Acid Tolerance between the Starved and Non-Starved Biofilms

**Figure 2:**
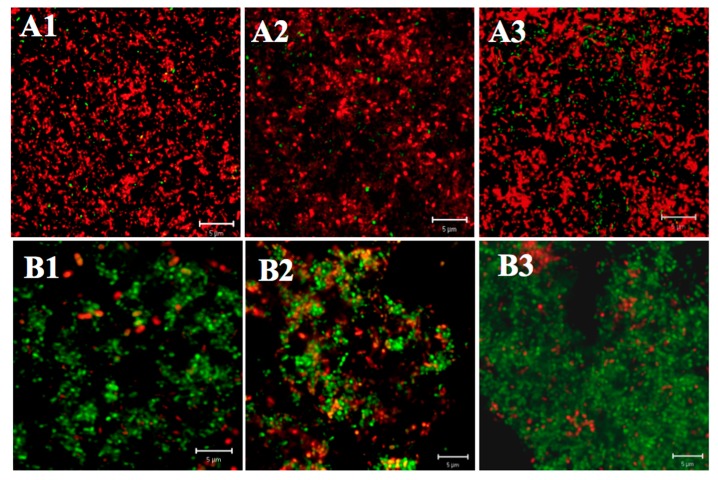
CLSM images of Live/Dead (SYTO-9 and PI) stained starved/non-starved biofilms after acid shock. Non-starved biofilm ((**A1**), ATCC25175; (**A2**), SM18; (**A3**), SM593); Starved biofilm ((**B1**), ATCC25175; (**B2**), SM18; (**B3**), SM593) (scale bars, 5 mm; magnification 630×).

As shown in Figure 2, the biofilm architecture of all three tested strains (ATCC 25175, SM18, and SM593) seemed comparatively less destructive in the starved group than in the non-starved group. Furthermore, the number of viable cell in each layer of starved biofilms was significantly larger than that of non-starved biofilms (Table 3) (all *p* < 0.05). Also, the comparisons of the viability of three different layers within each group (starved and non-starved groups) showed a similar trend as the ATR on acid tolerance.

#### 2.2.2. Differences in Acid Tolerance on Starvation among Three Strains

SM593 biofilm had a less likely loss of architectural integrity with significantly higher viability at each layer than *S. mutans* ATCC 25175 and SM18 in both starved and non-starved groups (all *p* < 0.05), whereas no significant difference was found between ATCC 25175 and SM18 biofilms (all *p* > 0.05) (Figure 2, Table 3).

### 2.3. Three-Dimensional Reconstructions

**Figure 3:**
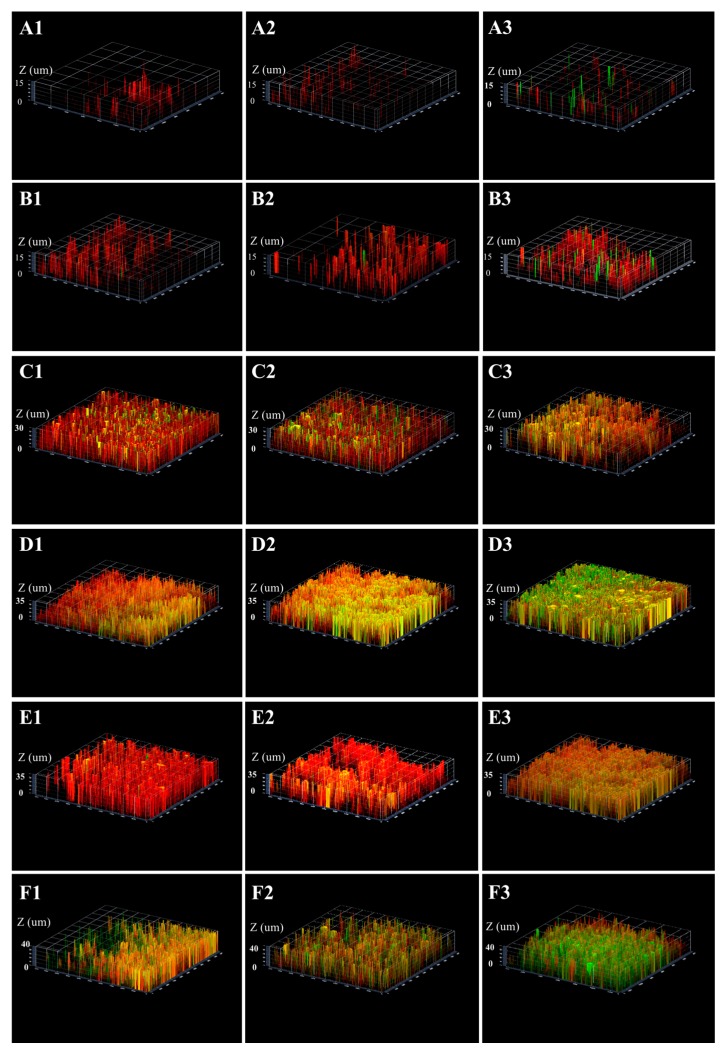
Three-dimensional architecture of *S. mutans* biofilms under different conditions. Young biofilm in non-adapted group (**A1**), ATCC25175; (**A2**), SM18; (**A3**), SM593; Young biofilm in acid-adapted group (**B1**), ATCC25175; (**B2**), SM18; (**B3**), SM593; Mature biofilm in non-adapted group (**C1**), ATCC25175; (**C2**), SM18; (**C3**), SM593; Mature biofilm in acid-adapted group (**D1**), ATCC25175; (**D2**), SM18; (**D3**), SM593 Non-starved biofilm (**E1**), ATCC25175; (**E2**), SM18; (**E3**), SM593; Starved biofilm (**F1**), ATCC25175; (**F2**), SM18; (**F3**), SM593.

The three-dimensional image reconstruction of each biofilm was obtained through the series CLSM images. As shown in Figure 3, for all three tested strains, the structures of acid-adapted (both mature and young biofilms) and starved biofilms were relatively flatter and thicker compared to gully shape structure of non-adapted and non-starved ones. Furthermore, in all cases, SM593 biofilm was less likely destructive and denser than that of ATCC 25175 and SM18.

## 3. Discussion

*S. mutans* is generally considered to be a principal etiological agent of dental caries. Variables of *S. mutans* have been investigated for their associations with the caries process. The ability of *S. mutans* to survive and grow in low pH environments is fundamental for its survival and eventual dominance in dental plaque, finally leading to caries. In other words, aciduricity of *S. mutans* plays a crucial role in the occurrence and development of dental caries and acts as an important virulence factor. Nevertheless, understanding on determination of aciduric virulence factor of *S. mutans*, especially for *S. mutans* in biofilm lifestyle, is limited. In the present study, *S. mutans* clinical strains isolated from caries-active (SM593) and caries-free (SM18) adults and the standard strain ATCC 25175 have been used to analyze the association between the aciduricity of strains and caries activity under different conditions (including biofilm age, ATR and starvation) to identify potential factors influencing the aciduricity of *S. mutans*.

The capacity of *S. mutans* to initiate caries via acid production from the metabolism of dietary carbohydrates [18] would be suicidal if not for its remarkable ability to tolerate acid, signifying a crucial aspect of its virulence [25]. In order to withstand these continual cycles of acid shock, *S. mutans* has evolved a repertoire of mechanisms that fall under two distinct categories; constitutive mechanisms and acid-induced mechanisms [11], also referred to as acid tolerance response (ATR) [7]. More specifically, the ATR of this microorganism is defined as the ability to adapt to acid stress by prior exposure to a low, sub-lethal pH of approximately 5.5 [25,26,27]. In the present study, based on the qualitative and quantitative analysis of CLSM data, we found that ATR could remarkably enhance the aciduricity of biofilms generated by biofilms SM593, SM18, and *S. mutans* ATCC 25175. This finding is consistent with those from previous studies demonstrating that the aciduricity of *S. mutans* in planktonic status could be enhanced by ATR and confirming the ability of *S. mutans* to induce ATR that enhanced its survival at a low pH [6,11,26]. Pre-acidification of sub-lethal pH 5.5 exposure for three hours prior to acid killing induced a stimulon which enhanced *S. mutans* survival at pH 3.0 [25,26,27]. Furthermore, the rapid acidification is also followed by the expression of numerous proteins, including chaperones and membrane proteins, all of which were necessary for cellular viability [28,29]. However, these possible mechanisms need to be confirmed by further study.

In addition, one of essential factors for viability is the regulation of formed biofilm. Previous studies investigated the effect of chlorhexidine gluconate on treating biofilms reported that the proportion of killed bacteria was much higher in young biofilms than in mature biofilms [30,31]. Likewise, results of the present study demonstrated that whether acid-adapted or non-adapted, the decrease in survivors and structure disruption were more significant in young biofilms than in the matures. It was indicated that mature biofilms might be more likely to prevent biofilm disruption and to offer stronger protection for cells under stress environments (such as acid killing), which were in keeping with findings from earlier works reporting that biofilm cells were more tolerant to environmental stress and antimicrobial agents than their young counterparts [9,32]. One of the possible explanations for this result is that mature biofilm is denser, more compact and complex compared with young biofilm, so it is harder for acid to penetrate. The finding supports that mature biofilms are more cariogenic than young ones. Therefore, besides the well-known fact that mature biofilm is more difficult to treat than young biofilm, removing dental plaque biofilm via cleaning techniques, such as regular toothbrushing and the use of dental flossing in early stage, is highly recommended.

Reportedly, starvation provided cells of *Escherichia coli* with an equivalent or even slightly higher level of acid tolerance than log-phase ATR [12]. Zhu et al. found that starvation could successfully induce the tolerance of planktonic *S. mutans* to acid shock at pH 3.8 [13]. Similarly, the results from the present study indicated that starvation enhanced acid tolerance of *S. mutans* in biofilm regardless of the genotypes of strains, supporting the speculation that oral bacteria in dental biofilm plaque is likely to be more tolerant to acid stress during starvation between meals [4]. This could partly explain the phenomenon in daily life that eating sugar or food high in sugar between meals increases the risk of dental caries [33].

On the other hand, in the presence or absence of adaptation and starvation, comparisons for any stage of biofilm between three tested strains of *S. mutans* indicated that SM593 biofilm was more acid-tolerant and induced a stronger ATR than SM18 and ATCC 25175 biofilms. This result is consistent with the finding that different strains of *S. mutans* exhibited different degrees of low pH tolerance [34], and also supports that of our previous study, which demonstrated that SM593 had stronger aciduricity than SM18 in planktonic [24]. Biofilm was proposed to play a crucial role in acid tolerant of *S. mutans* [34]. In our previous study, we also found that SM593 exhibited a superior capacity to form more complex biofilm than SM18 and ATCC 25175 [35]. Therefore, we speculate that more complex structure of biofilm and greater ability of SM593 to form biofilm could partly explain a stronger capability of SM593 biofilm to tolerate low pH and grow compared with SM18 biofilm and ATCC 25175. Moreover, *S. mutans* genotypes from caries-free and caries-active individuals also differed in their ability to withstand acid challenge, and we extrapolate that the differences in caries susceptibility may be attributed to the colonization of specific strain, such as SM593. Whereas, it is worthwhile to note that there was no statistical difference in viability between SM18 and ATCC 25175. This may implicate that the virulence of standard strain ATCC 25175 was similar to that of SM18.

For each strain, the viability was highest in the middle biofilm layer while lowest in the inner layer. The viability varied from inner layer to outer layer because bacterial cells at different locations within a biofilm might not sense the same degree of pH stress simultaneously, and nutrient availability might be restricted when biofilm became thicker. This could be partly explained by the facts as reported in earlier reports [36,37,38]. Viable cells of the inner layer were least under different conditions, a plausible explanation of which probably lying in that relatively inadequate nutrition might slow their growth. Additionally, cells’ metabolites might be detained in the inner layer, which possibly affected their growth as well. On the contrary, there were significantly more viable cells in the middle layer, where there was a more anaerobic condition and higher supply of nutrition, and inner and outer biofilm layers protected the cells in the middle biofilm layer against adverse stimulation from the environment stress. It was indicated that middle layer is likely the most suitable layer for bacteria to survive. The lower viability in the outer layer can be explained by the hypothesis that bacteria in this layer were directly exposed to adverse environmental stimulations which resulted in severe damage, although there was abundant nutrition for their growth. In addition, a similar viability distribution of the three layers in these strains of *S. mutans* also suggest that standard strain ATCC 25175 and clinical strains SM593 and 18 may have similar effect on regulating biofilm structure.

Nevertheless, the limitation of the present study focusing only on single-species biofilms of *S. mutans* should be noted. Multi-species biofilm formed by several cariogenic microorganisms, which is more close to natural dental plaque biofilms, need to be discussed in further study to complement the result of the current study.

## 4. Materials and Methods

### 4.1. Bacterial Strains and Culture Conditions

All experiments described herein were approved by the component local authorities. All procedures were done in agreement with National Institutes of Health guidelines. SM593 was isolated from caries-active adult who had multiple active carious lesions (the number of decayed and filled teeth (DFT) = 10, no missing tooth, and three non-restored cavities). SM18 was isolated from caries-free adult (the number of decayed, missing, and filled teeth (DMFT) = 0) [23]. *S. mutans* standard strain ATCC 25175, friendly provided by State Key Laboratory of Oral Diseases, Sichuan University (Chengdu, China), was applied as reference. Bacteria were cultured in tryptone-polypeptone-yeast extract (TPY) (Oxoid, Hampshire, England, UK) at 37 °C in a controlled anaerobic environment (80% N_2_, 10% CO_2_, and 10% H_2_). Pure cultures of each test strain were obtained and resuspended in fresh TPY with an optical density (OD) of 1.0 at 630 nm (approximately 1 × 10^8^ cells/mL) for following experiments.

### 4.2. Formation of Young and Mature Biofilms

*S. mutans* biofilms were formed on sterile polystyrene plastic sheets (1 mm × 1 mm, Dow Corning, Germany) which were immersed in Petri dishes containing 19 mL of TPY and 1 mL of resuspended bacterial suspension. The sheets were then incubated in an anaerobic chamber at 37 °C. The young and mature biofilms were formed on the plastic sheets after 3 h and 20 h incubation, respectively.

### 4.3. Acid-Adaptive Response of Young and Mature Biofilms

After the formation of young and mature biofilms, the biofilm sheets were rinsed with phosphate-buffered saline (PBS; pH = 7.0) to remove non-adherent cells and then randomly divided into acid-adapted and non-adapted groups. The non-adapted group was immersed directly in TPY medium of pH 3.0 for 30 min, whereas the acid-adapted group was first immersed in TPY medium of pH 5.5 for 3 h and then into the medium of pH 3.0 for 30 min.

### 4.4. Acid Shock of Starved/Non-Starved Biofilms

After 20 h of biofilm formation, all sheets were rinsed in 1 mL PBS (pH = 7.0) to remove non-adherent cells, and then divided into non-starved and starved group randomly. The biofilms in the non-starved group were incubated in 20 mL TPY medium for 24 h and then immersed in TPY medium of pH 3.0 for 30 min. The biofilms of the starved group were immersed in PBS for 24 h and then into TPY medium of pH 3.0 for 30 min.

### 4.5. Fluorescence Staining for Biofilms (Viability Staining)

The Live/Dead BacLight Bacterial Viability Kit (L-7012, Molecular Probes, Eugene, OR, USA) contains SYTO-9 and propidium iodide (PI), which both stain nucleic acids. The kit was used to implement fluorescence staining for biofilms, following the manufacturer’s instructions. Briefly, both SYTO-9 and PI diluted by distilled water at a ratio of 1.5:1000 and then thoroughly oscillated. Viable bacterial cells were stained with SYTO-9 (green), and bacterial cells with damaged membranes were stained with PI (red). The biofilms were removed from the culture media and washed with PBS to remove non-adherent cells. Each biofilm was immersed in 200 μL of the corresponding stain, incubated for 15 min in the dark at room temperature, and then rinsed with 200 μL of PBS. Next, *p*-phenylenediamine (5 mM) in PBS was added on top of the biofilm, which was then covered with a glass cover slip and viewed with CLSM.

### 4.6. CLSM Observation of Biofilms and Image Analysis

The stained biofilms were examined using a Zeiss LSM 510 CLSM (Carl Zeiss Microscopy, Germany). For each experiment, all parameters, including laser intensity, background level, contrast and zoom, were maintained at the same level. At 5 μm intervals, a series of optical sections were acquired from the surface through the vertical axis of the specimen using a computer-controlled motor drive. Each biofilm was scanned at four randomly selected positions, and three series of optical sections were generated at each position. Image stacks were analyzed using the Java-based image analysis program Image J (Version 1.38, National Institutes of Health, Bethesda, MD, USA). Image J was used to count green (live cells) and red (dead cells) areas of the biofilms. The three-dimensional architecture of the biofilm was visualized with Zeiss LSM software. The percentage of live bacterial cells (viability) was calculated as a result of green areas/(red areas + green areas).

### 4.7. Statistical Analysis

The data were statistically analyzed using Statistical Package for Social Sciences (SPSS, version 20.0 software (Chicago, IL, USA). Results of the cell viability were reported as mean ± SD. One way analysis of variance (ANOVA) and t-test were used to compare means among three strains (SM593, SM18 and ATCC 25175) and between two different conditions (such as, young biofilm vs. mature biofilm, acid-adapted vs. non-adapted, starved vs. non-starved), respectively. *p* < 0.05 was considered significantly different. Each experiment included six samples. Each assay was carried out in triplicate.

## 5. Conclusions

In conclusion, both ATR and starvation can enhance the aciduricity of biofilm formed by *S. mutans* regardless of strains. In addition, mature biofilm is more acid-resistant than young biofilm. Furthermore, SM593 from caries-active individuals exhibits a higher aciduricity than SM18 from caries-free individuals and standard strain ATCC 25175 in all cases, which may contribute to a better understanding of the differences in caries activity observed among *S. mutans*-infected individuals. The current study provides a potential and novel way to detect the cariogenic mechanism of *S. mutans* and to develop measures for caries prevention and management, especially for individuals with high risk of caries.

## Figures and Tables

**Table 1:** The viability of three strains in different layers of young biofilms (mean ± SD).

Strains	Outer		Middle		Inner	
	Non-Adapted	Acid-Adapted	Non-Adapted	Acid-Adapted	Non-Adapted	Acid-Adapted
ATCC25175	3.15 ± 0.67	11.12 ± 0.85	13.27 ± 1.17	24.17 ± 1.01	4.34 ± 0.91	11.85 ± 0.75
SM18	2.56 ± 0.79	10.91 ± 0.92	12.45 ± 0.95	21.42 ± 0.95	3.23 ± 0.96	10.99 ± 0.81
SM593	7.72 ± 0.57	15.41 ± 0.96	22.56 ± 0.87	42.37 ± 0.44	8.51 ± 0.91	15.65 ± 0.65

**Table 2:** The viability of three strains in different layers of mature biofilms (mean ± SD).

Strains	Outer		Middle		Inner	
	Non-Adapted	Acid-Adapted	Non-Adapted	Acid-Adapted	Non-Adapted	Acid-Adapted
ATCC25175	12.26 ± 0.22	18.43 ± 1.23	29.72 ± 0.97	49.88 ± 0.87	12.49 ± 0.74	19.76 ± 0.76
SM18	11.48 ± 0.75	17.52 ± 1.11	28.71 ± 1.04	47.79 ± 0.83	13.42 ± 0.85	18.52 ± 1.16
SM593	16.51 ± 0.96	31.19 ± 1.06	38.73 ± 0.74	68.75 ± 0.95	17.31 ± 0.76	34.45 ± 1.36

**Table 3:** The viability of three strains in different layers of starved biofilms (mean ± SD).

Strains	Outer		Middle		Inner	
	Non-Starved	Starved	Non-Starved	Starved	Non-Starved	Starved
ATCC25175	13.36 ± 1.05	21. 67 ± 0.66	27.21 ± 1.05	47.88 ± 0.87	14.77 ± 0.76	23.58 ± 0.88
SM18	11.39 ± 1.10	22.47 ± 0.91	26.16 ± 1.15	47.24 ± 1.25	12.14 ± 0.21	23.28 ± 1.07
SM593	21.47 ± 1.30	41.59 ± 0.99	39.28 ± 0.89	61.31 ± 0.76	23.11 ± 0.31	43.35 ± 0.92

## Abbreviations

ATCC	American Type Culture Collection
ANOVA	One way analysis of variance
ATR	Acid tolerance response
CLSM	Confocal laser scanning microscopy
DMFT	decayed, missing, and filled teeth
DFT	decayed and filled teeth
MS	*Mutans Streptococcus*
OD	Optical density
PBS	Phosphate-buffered saline
PCR	Polymerase chain reaction
PI	Propidium iodide
TPY	Tryptone-polypeptone-yeast extract
SD	Standard deviation
SPSS	Statistical Package for Social Science
